# Bilateral gain control; an “innate predisposition” for all sorts of things

**DOI:** 10.3389/fnbot.2014.00009

**Published:** 2014-02-25

**Authors:** Nicholas Wilkinson, Giorgio Metta

**Affiliations:** ^1^iCub Facility, Istituto Italiano di TecnologiaGenova, Italy; ^2^Centre for Robotics and Neural Systems, University of PlymouthPlymouth, UK

**Keywords:** bilateral symmetry, gain control, innate predisposition, embodiment, beamforming, attention, “like me”, human–robot interaction

## Abstract

Empirical studies have revealed remarkable perceptual organization in neonates. Newborn behavioral distinctions have often been interpreted as implying functionally specific modular adaptations, and are widely cited as evidence supporting the nativist agenda. In this theoretical paper, we approach newborn perception and attention from an embodied, developmental perspective. At the mechanistic level, we argue that a generative mechanism based on mutual gain control between bilaterally corresponding points may underly a number of functionally defined “innate predispositions” related to spatial-configural perception. At the computational level, bilateral gain control implements beamforming, which enables spatial-configural tuning at the front end sampling stage. At the psychophysical level, we predict that selective attention in newborns will favor contrast energy which projects to bilaterally corresponding points on the neonate subject's sensor array. The current work extends and generalizes previous work to formalize the *bilateral correlation model* of newborn attention at a high level, and demonstrate in minimal agent-based simulations how bilateral gain control can enable a simple, robust and “social” attentional bias.

## 1. Introduction

The empirical position is, to be sure, in agreement with the nativistic on a number of points—for example, that local signs of adjacent places on the retina are more similar than those farther apart and that the corresponding points on the two retina are more similar than those that do not correspond. Helmholtz (1968)

Psychophysical studies have demonstrated quite sophisticated spatial-configural perception in newborn babies. See for example Johnson et al. (1991); Slater and Kirby (1998); Farroni et al. (2000); and Streri et al. (2013a). Such findings have often been interpreted in terms of functionally defined “innate predispositions” (Morton and Johnson, 1991; Spelke, 1998; Streri et al., 2013b), which are seen as providing the “biological basis” of perceptual and social development (Johnson and Morton, 1991; Johnson, 2003). Other researchers have suggested that conspecifics are identified as “like me” via perceptuomotor resonance with internal representations of the self, developed prenatally through self-exploration and proprioception (Sugita, 2009; Pitti et al., 2013). Some have even proposed that an innate precursor of the mirror neuron system underlies newborn sociality (Meltzoff and Decety, 2003; Lepage and Théoret, 2007). Here we extend and generalize previous work on newborn face preference (Wilkinson et al., in press), to outline a high level formalization of a novel perspective in which *embodied sampling biases* provide “innate information” about space, configuration, and “like-me-ness.”

Specifically, the sampling bias under focus in the current paper is *bilateral* sensor distribution and integration. Many authors have noted the important role of multimodal correlation in social interaction and perceptual learning (e.g., Bahrick et al., 2004; Sai, 2005). Less attention has been paid to the implications of intramodal bilateral correlations from the social and behavioral perspective, though bilateral sensory interaction has been intensively studied in its own right (see “Bilateral mutual gain control” below). Here we hypothesize a functional relationship between bilateral interaction and spatial-configural perception in newborns. A simplified formal model explains how bilateral mutual gain control can enable spatial-configural perceptual distinctions analogous to some of those observed in human newborns. Agent-based simulations address minimal analogs of visual perception of “size constancy” (Granrud, 1987; Slater et al., 1990), visual and audio-visual face perception (Goren et al., 1975; Johnson et al., 1991; Sai, 2005), and the dependency of face perception and social learning on infant directed alignment, or “direct gaze” (Farroni et al., 2002; Guellai and Streri, 2011).

In section 2, we present a case for bilateral mutual gain control as a general aspect of intersensory integration, based on the existing literature, and recruit the array signal processing formalism of beamforming (Naidu, 2001) as a way of describing spatial-configural attention and orienting. In section 3, we define a minimal formal model to describe how bilateral gain control can generate an overt attentional preference for the “like me.” In section 4, we report results of simulations based on this model. Finally, we discuss the relevance and scope of our theoretical findings, and offer some concluding remarks.

## 2. The newborn as a multimodal, bilateral sensor array

Gibson (1966) argued that the sensing body should be characterized as a multimodal sensor array. In any sensing process, the front line is the sampling regime adopted; neural/computational processing can only process that which has been sampled. It is therefore impossible to meaningfully characterize sensory input to the nervous system during active behavior without considering the physical embodiment of the sensor array (Towal et al., 2011). The vertebrate sensor array is structured in a more or less bilaterally symmetric manner. A growing consensus points to mutual gain control as a fundamental feature of bilateral interactions (e.g., Li and Ebner, 2006; Ding et al., 2013; Schmidt, 2013; Wunderle et al., 2013; Xiong et al., 2013). Mutual gain control is one mode of enacting *beamforming* (e.g., Westermann, 2003; Ma, 2013), a standard technique for selective tuning in sensor array technology. In this section, we unpack this analogy between selective attention in newborn infants and selective tuning in sensor array theory. First, we review the neurobiological literature on bilateral mutual gain control and the relationship of gain control to attention. This sketches a plausible neural substrate for the current model, which we term the “bilateral correlation model” (“BCM”) of newborn attention. We then briefly introduce beamforming and explain how it is related to bilateral gain control and attention.

### 2.1. Bilateral mutual gain control and sensory attentional gating

#### 2.1.1. Bilateral symmetry structures vertebrate physiology

The bilateral structure of the brain and body is aligned and integrated according to symmetric correspondence at many stages of sensory and motor processing, an architecture perhaps most clearly demonstrated by the corpus callosum (Iwamura, 2000; Li and Ebner, 2006), which links corresponding bilateral points in the brain. Tactile stimulation of one hand causes bilateral cortical activation at corresponding somatotopic points (Hansson and Brismar, 1999). Binocular cross-correlation is widely thought to underly stereo vision (Banks, 2007; Filippini and Banks, 2009). Binaural cross-correlation informs orienting and looking behavior in neonates (Mendelson et al., 1976; Jiang and Tierney, 1996; Furst et al., 2004), and the foetus is capable of auditory orienting *in utero* (Voegtline et al., 2013). From an aesthetic perspective, the “sweet spot” region of binaural synchrony is manipulated by sound engineers to deliver the most enjoyable and engaging listening experience (Theile, 2000; Bauck, 2003), suggestive of a more general multimodal link between bilateral correlation, arousal and “liking.”

#### 2.1.2. Neural gain control implements selective attention

Neural *gain control* can implement multiplicative interactions *in vivo* (Rothman et al., 2009). Gain control acts like an amplifier, or “gate” for input signals. Gain control is widely thought to mediate selective attention (Hillyard et al., 1998; Salinas and Sejnowski, 2001; Aston-Jones and Cohen, 2005; Reynolds and Heeger, 2009; Feldman and Friston, 2010; Katzner et al., 2011; Sara and Bouret, 2012), and has been mechanistically linked to ascending projections from neuromodulatory hubs and the sympathetic nervous system (Aston-Jones and Cohen, 2005; Voisin et al., 2005; Sara, 2009; Fuller et al., 2011). Presynaptic sychrony can also modulate postsynaptic gain in a feedforward fashion (Huguenard and McCormick, 2007; Womelsdorf and Fries, 2007; Gotts et al., 2012). Gain control has been formally equated with the modulation of Bayesian *precision* in probabilistic generative modeling (Feldman and Friston, 2010; Moran et al., 2013).

#### 2.1.3. Bilateral mutual gain control

There is extensive evidence that mutual gain control is an important aspect of binocular (Ding and Sperling, 2006; Meese and Baker, 2011; Ding et al., 2013), binaural (Kashino and Nishida, 1998; Ingham and McAlpine, 2005; Steinberg et al., 2013; Xiong et al., 2013), and bitactile (Hansson and Brismar, 1999; Li and Ebner, 2006) interactions in various species. Interhemispheric interactions via corpus callosum are well described by a gain control relation (Li and Ebner, 2006; Schmidt, 2013; Wunderle et al., 2013). Thus mutual gain control is the most plausible general framework for bilateral sensory interaction, though many particulars are likely to exist at a more detailed level. Given this organization, a behavioral preference for stimuli which induce strong correlations between corresponding points is practically inevitable.

#### 2.1.4. Bilateral gain control in the neonate

Studies examining prestereoptic binocular vision in human infants have produced conflicting results, and have not included neonatal subjects (Shimojo et al., 1986; Brown and Miracle, 2003; Kavšek et al., 2013). The maturity of binocular gain control circuitry in human newborns is therefore unknown. In the rhesus macaque, considered a good model for the human visual system, binocular circuitry is quite mature in neonates (Rakic, 1976; Horton and Hocking, 1996), and responses are limited by low monocular sensitivity rather than binocular immaturity (Chino et al., 1997). Binaural integration is also functioning, if not entirely mature, in neonates (Furst et al., 2004; Litovsky, 2012). The BCM assumes that newborn bilateral integration is a qualitatively similar, if immature, version of that in adults, but does not prescribe the precise transform; many variations on mutual gain control are possible. In adults, bilateral interactions are modulated by mono and stereo normalization (Carandini et al., 1997; Moradi and Heeger, 2009; Carandini and Heeger, 2011), but the nature of normalization in the newborn human is, as far as we know, unknown.

### 2.2. The thalamus—an architectural hub for attentional gating and bilateral alignment

#### 2.2.1. Bilateral alignment in the thalamus

The thalamus embodies architectural alignment of the signals from bilaterally corresponding sensors (Jones, 1998). For example, the *lateral geniculate nucleus* (henceforth “LGN”), is comprised of six layers. Connections from contralateral nasal retina project to layers 1, 4, and 6, while ipsilateral temporal retina projects to layers 2, 3, and 5. All of these layers are precisely in-reference with respect to retinal receptive fields. This architecture forms a structural basis for precise binocular receptive fields in efferents such as primary visual cortex. Bilateral auditory (Wrege and Starr, 1981; Fitzpatrick et al., 1997; Ingham and McAlpine, 2005; Krumbholz et al., 2005) and tactile (Mountcastle and Henneman, 1949; Emmers, 1965; Davis et al., 1998; Coghill et al., 1999) pathways also converge in the thalamus and earlier.

#### 2.2.2. Sensory gain control and attention in thalamus

An active role for the thalamus in attention has long been theorized (Clark, 1932; Crick, 1984), and evidence supporting these suggestions is accumulating (Varela and Singer, 1987; Sillito et al., 1994; Sherman and Guillery, 2006; Sherman, 2007; Saalmann and Kastner, 2011; Ward, 2013). The thalamus is strongly associated with gain control (Saalmann and Kastner, 2009). LGN receives only about 10% of its input from retina, with the other 90% constituted in approximately equal proportions by cholinergic projections from the parabrachial nucleus of the brainstem, inhibitory control from the thalamic reticular nucleus, and feedback connectivity from layer 6 in striate cortex (Saalmann and Kastner, 2009). Recently, layer 6 in visual cortex has been shown to mediate gain control of superficial layers (Olsen et al., 2012; Vélez-Fort and Margrie, 2012). This extensive modulatory network effects gain modulation in LGN, thereby gating visual input to the cortex (Sherman, 2007; Saalmann and Kastner, 2011; Lien and Scanziani, 2013). Corticofugal feedback also modulates gain control in auditory thalamus (Grothe, 2003; He, 2003; Zhang et al., 2008).

### 2.3. Beamforming, orienting, and motor attention

**Figure 1:**
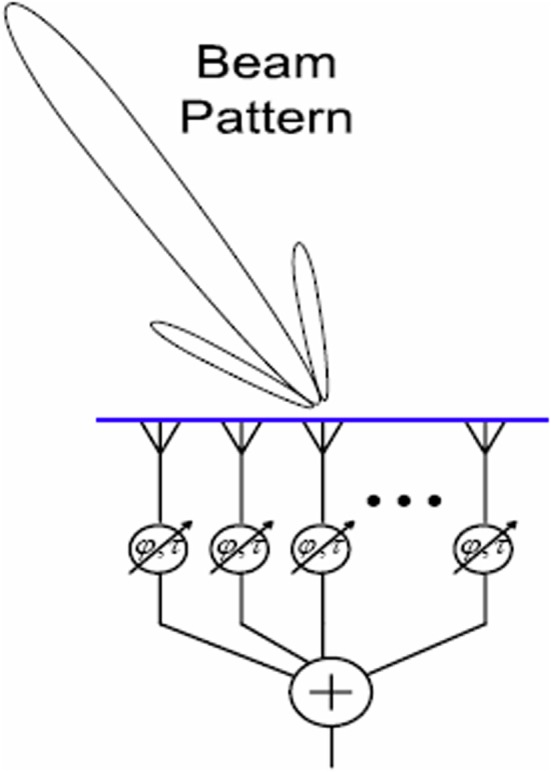
**A schematic depiction of beamforming**. The angle of arrival to which the array is tuned is represented by the “beam” projected onto space. This may be steered by adding delays at the integration step, or by physically turning the array.

Beamforming, a form of spatial filtering, is a technique for manipulating the *spatial tuning* of a sensor array (Naidu, 2001). Applications include astronomy (e.g., the SCUBA-2 project^1^), neuroimaging (Van Veen and Buckley, 1988; Siegel et al., 2008), “smart” audio technology (Kellermann et al., 2012; Sun et al., 2012), and network communication (Litva and Lo, 1996; Lakkis, 2012). The mathematical essence of beamforming is maximization of constructive interference between the signals from an array of sensors. Integrating the signals from an array of spatially distributed sensors creates a “beam,” or preferred angle of arrival, for incoming signals. Mutual gain control is one possible integration function e.g., Ma (2013). When the signals from all the sensors are temporally aligned, constructive interference is maximized and the input signal is faithfully reproduced. Adding differential delays to the sensor inputs, or physically turning the array, can rotate this beam in space, so that sources at particular locations (e.g., a mobile phone) can be targeted, whilst noise from elsewhere is tuned out; a kind of technological “selective attention.” See Figure 1 for a schematic depiction.

For example, in delay-and-sum beamforming, a large set of delays is provisionally added to the signals from the array. The particular delay(s) which maximizes the constructive interference with a reference sensor (i.e., maximizes the combined signal from the whole array) is identified. This optimum delay is proportional to the angle of arrival of the signal and may be calculated as;

(1) $$\Delta (t_{i})=\frac{(i-1)d\cos\theta }{c},i=1,2\ldots n$$

Where *d* is inter-sensory distance, *c* is the traveling speed of the signal (e.g., the speed of sound), θ is the angle of arrival of the signal, and *i* represents the position of the sensor concerned relative to the reference sensor. More than two sensors can of course be used. In practive, θ is usually not known.

#### 2.3.1. Audio beamforming

Beamforming is mathematically similar to the widely accepted notion of sound localization based on inter-aural time difference (“ITD”) (Jeffress, 1948; Fitzpatrick et al., 2000, 2002; Joris and Yin, 2007; Hartmann et al., 2013). Binaural hearing aids employ beamforming to give directional selectivity, allowing the device to focus on sound sources at the auditory midline (Greenberg and Zurek, 1992; Kompis and Dillier, 2001; Westermann, 2003; Ma, 2013). A recent study found that auditory adaptive coding mechanisms primarily target sources near the interaural midline (Maier et al., 2012), suggestive of a “special” attentional status for midline sources. Audio beamforming is fundamental to technological approaches to the “Cocktail Party Problem” (Cherry, 1953; Haykin and Chen, 2005).

#### 2.3.2. Motor attention and beamforming

Adding the delay Δ(*t*_*i*_) steers the angle of arrival to which the array is tuned, and is equivalent to physically turning the array. Physically turning the array is analogous to the psychological concept of orienting or *overt attention*. Adding delays to “virtually” orient the array is analogous to “covert attention.” Covert attention and overt attention are thought to be tightly linked (Eimer et al., 2005; de Haan et al., 2008), though appear to be mediated by different cellular networks (Gregoriou et al., 2012). Physically turning the array provides the basis for the motor side of selective attention in the current model. A movement equivalent to the delay is enacted in order to physically align the array with the source. We deal only with the overt orienting case here. Thus Δ(*t*_*i*_) corresponds to a motor command, rather than an internal delay. Here we reference this difference by the term “active beamforming.” This may seem a little topsy-turvy, as beamforming was to a large extent designed to avoid the need for slow and energy hungry mechanical orienting of the array, but we believe it conveniently expresses the underlying continuity between mechanical and “virtual” manipulation of alignment.

#### 2.3.3. Visual beamforming

The speed of light renders delays virtually undetectable. Light's beamlike propagation and the design of visual optics provide direction selectivity *a priori*. Thus visual beamforming is not standard practice, and the terminology adopted here may thus be non-standard. However, the mathematical concept at the heart of beamforming—maximizing constructive interference between sensors—applies equally to the visual case in the current model. As the underlying sensory computations are the same between vision and audition, we adopt the continuous terminology “visual beamforming.”

**Figure 2:**
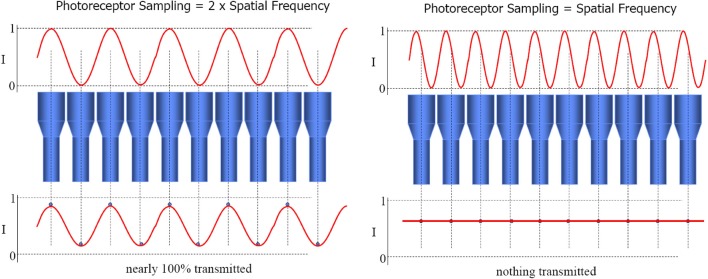
**The loss of the sinusoidal signal when signal frequency equals sensor distribution frequency constitutes morphological resonance**. Constructive interference, and hence combined output of the array, is maximized in this case. Image slides courtesy of Austin Roorda, UC Berkeley http://roorda.vision.berkeley.edu.

The beamforming technique provides an array tuned to two different flavors of stimulus. Firstly, *one source* that lies on the intersection of the sensors' lines of sight (as just described for audio beamforming). Secondly, *multiple sources*, one on each line of sight. This yields tuning to particular *configurations* of multiple signal sources, simply through spatial resonance between the configuration of transmitters and the configuration of receivers. In the case of sensors with parallel directional tuning, this “preferred” configuration is the *same configuration* as that of the sensor array itself. Thus it functions as a “like me” configuration detector. This is the special case of Nyquist–Shannon sampling theorem, wherein signal frequency equals sensor distribution frequency, causing maximal constructive interference between the combined signals. See Figure 2. Prior to the development of convergent stereopsis, newborn binocular alignment is (approximately and noisily) parallel (Thorn et al., 1994), though this depends on what the infant is looking at (Slater and Findlay, 1975).

Multiple source signals often occur in the context of problems in sensor array theory, indeed it is this sensitivity that is targeted by signal jamming techniques (Poisel, 2011). However, multiple transmitter, multiple receiver set-ups are widely used in wireless communication (termed “MIMO” multiple-input and multiple-output beamforming e.g., Raleigh and Cioffi, 1998) and may be highly relevant in the case of biological vision. Eyes are a particularly important example of a multiple visual signal source (Gliga and Csibra, 2007). Single transmitter and multiple transmitter beamforming specifies two ways in which a scene can be “like me” to a newborn infant; it can “fit” my sensor array by containing *single sources* which lie on the intersections of my bilateral lines of sight, and it can “fit” my sensor array by containing *multiple* sources which independently occupy both the lines of sight of a bilateral sensor pair. To our knowledge, this *functional relationship* between “visual beamforming,” spatial-configural vision and “like-me” perception in the newborn is a novel proposal. However, the underlying mechanisms proposed are not; they are just those of standard binocular circuitry and established communications technology.

## 3. A formal model

In this section, we define a simplified model world in which to formalize our arguments and demonstrate the resultant effects in minimal computational simulations.

### 3.1. A simple world

**Figure 3:**
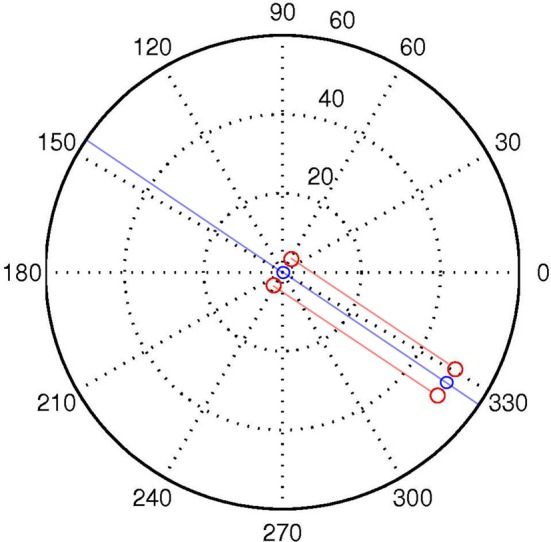
**A logpolar World, containing two “agents,” one located at the origin, the other at distance of ρ = 50, and aligned with (“looking at”) the other agent at the origin**. The red dots correspond to the positions of the sensors of each agent. The semi-transparent red lines correspond to the LoS of the central visual sensors for each “eye.” The semi-transparent blue line demarks the auditory midline, or line of equidistance from the two auditory sensors. This corresponds to the “beam” projected in beamforming. The blue circles represent the “mouth” of the agents.

Let us first define a discrete World *W* with two spatial dimensions *P*, Θ, and time *T*. *P* and Θ refer to the location (in polar coordinates) in space of arbitrary quanta each with one binary degree of freedom *C*. See Figure 3. We use lowercase letters to denote specific values of capitalized variables, and to denote specific members of capitalized sets. Specifically, *W* is the set of all individual discrete World points *w*, each of which is characterized by four state variables ρ, θ, τ, *c*.

(2) $$\begin{array}{l} \;\forall w\{w\in W\}\\ w=<\rho ,\theta ,\tau ,c> \end{array}$$

*C* corresponds to the presence (“1”) or absence (“0”) of a visual signal source. Within this World, a subset of points *w* have *c* = 1 (indicating a visual signal source). We denote this set *W*_*C*_ ⊆ *W*;

(3) $$\forall w\{w\in {\bar{W}}_{C}:w_{c}\;=\;1\}$$

### 3.2. Building an agent

#### 3.2.1. A single visual sensor

Let us introduce a binary visual sensor, which occupies one space/quanta *w* in the World *W*. It has a tight beam, perfectly straight line of sight. We treat the travel time of light as zero. It is convenient to place the sensor at the origin, such that for any given location in the World *w* that location is on the line of sight if and only if *w*_θ_ = Sensor_θ_. The line of sight is then a set *L* containing all locations *w* in the World satisfying

(4) $$\forall w\{w\in L:\theta_{w}=\theta_{\text{sensor}}\}.$$

The sensor output *S*_Eye_ is 1 just in case its line of sight contains at least one point *w* where *w*_*c*_ = 1, that is if *L* intersects *W*_*C*_, otherwise it outputs zero. This may be viewed conveniently as a logical disjunction (OR gate) with the entire line of sight as input, which we will denote *S* = ∨*L*. The presence of a sensor at a point *w* sets *w*_*c*_ = 1, i.e., the sensor is also a visual signal source.

#### 3.2.2. A binocular agent

Let us place two visual sensors in the World, oriented in parallel and separated by an inter-sensory distance Φ. We refer to this sensor pair as “eyes” for readability. They are connected via a logical AND gate. The output of this AND operation is denoted *Ṡ*_Eyes_ and may be described in full as

(5) $${\dot{S}}_{\text{Eyes}}=\text{\&}(\vee {\text{L}}_{\text{Eye1}},\vee {\text{L}}_{\text{Eye2}}).$$

This is a minimal analogy of mutual gain control, the AND operation being equivalent to multiplication in the binary case. It is convenient to view *Ṡ*_Eyes_ as a single “meta-sensor” with a double aperture and a “forked” line of sight. Let us denote this forked LoS ${\dot{L}}_{\text{Eyes}}$, of which *L*_Eye1_ and *L*_Eye2_ are subsets. *Ṡ*_Eyes_ = 1 if and only if both *L*_Eye1_ and *L*_Eye2_ intersect *W*_*C*_.

#### 3.2.3. Vergence

Vergence eye movement changes the distance separating the visual axes (i.e., the LoS of the eyes), here denoted $\vec{\Phi }$, over depth, according to;

(6) $${\vec{\Phi }}_{\rho }=\Phi -\rho \tan\theta$$

where ρ is depth, and θ is vergence angle in radians. Positive θ corresponds to convergence and negative to divergence. $\vec{\Phi }$ and Φ are measured in the same units as ρ. With no vergence/parallel axes (i.e., θ = 0), this reduces to $\vec{\Phi }$ = Φ at any depth. In the current model the sensors are assumed to be aligned in parallel, to keep things simple.

#### 3.2.4. A dyad of binocular agents

In order to show how this set-up enables “like me” detection, let us introduce a second agent into the World (as in Figure 3). At this stage, it is useful to adopt the term “transceiver,” which references a mechanism which is both a transmitter and a receiver. This terminology becomes useful in the dyadic case, as one interactor's sensor is the other's signal. If we place the two agents in alignment, their pairs of visual transceivers mutually satisfy one another's sensory condition *Ṡ*_Eyes_ = 1 (Figure 3). However, if we translate either agent relative to the other, they no longer stimulate one another in this way. In order to see the other agent when it is not directly in front, a wider visual field of view is required.

#### 3.2.5. Wide field of vision

Let us define an agent with numerous copies of the sensor arrangement just described, analogous to a “pin-hole camera.” Each agent then has two wide angle sensors (“eyes”), while each “eye” consists of many individual light sensors (“rods”) with tight beam lines of sight; a *nested* sensor array. Formally, this may be represented simply by expressing the above description in terms of pointwise vector or matrix operations. The calculations are just the same, but repeated in parallel across the visual field. With this wider field of vision (“FoV”), an agent may see things not only directly in front, but also off to the side. The *spatial* offset from center may then be used to guide orienting. Thus we define each eye as a vector of individual sensors;

(7) $$\begin{array}{l} {\vec{S}}_{\text{Eye1}}=[S_{\text{Eye1},1},S_{\text{Eye1},2},S_{\text{Eye1},i},...S_{\text{Eye2},n}]\\ {\vec{S}}_{\text{Eye2}}=[S_{\text{Eye2},1},S_{\text{Eye2},2},S_{\text{Eye2},i}...S_{\text{Eye2},n}] \end{array}$$

and corresponding points (⇔) according to;

(8) $$\begin{array}{l} S_{\text{Eye1},1}\Leftrightarrow S_{\text{Eye2},1},\\ S_{\text{Eye1},i}\Leftrightarrow S_{\text{Eye2},i}\ldots \end{array}$$

For each corresponding bilateral sensor pair, there is an integration node *Ṡ*_Eyes,*i*_, yielding a vector of integration nodes ${\vec{\dot{S}}}_{\text{Eyes},1\ldots i}$. If any of these are 1, that is if the agent encounters binocular correlations in its field of view, then it goes in to “align” mode (see “Movement” below). The extent of the FoV for a visual sensor was arbitrarily set to be 0.1π, but other values could be used. Sampling resolution σ within this FoV was set to 0.0005 radians, but other values could be used.

However, there is connection to audition via $\ddot{S}$ only for the “foveal” sensor pair at the center of the field of vision *S*_Eye1,Center_ ⇔ *S*_Eye2,Center_ (see “Inter-modal integration” below). Peripheral occurrences of *Ṡ*_Eyes,*i*_ = 1 are used only for orienting, which brings the signal to align with *S*_Eye1,Center_ ⇔ *S*_Eye2,Center_.

#### 3.2.6. Audio sensing and signalling

**Figure 4:**
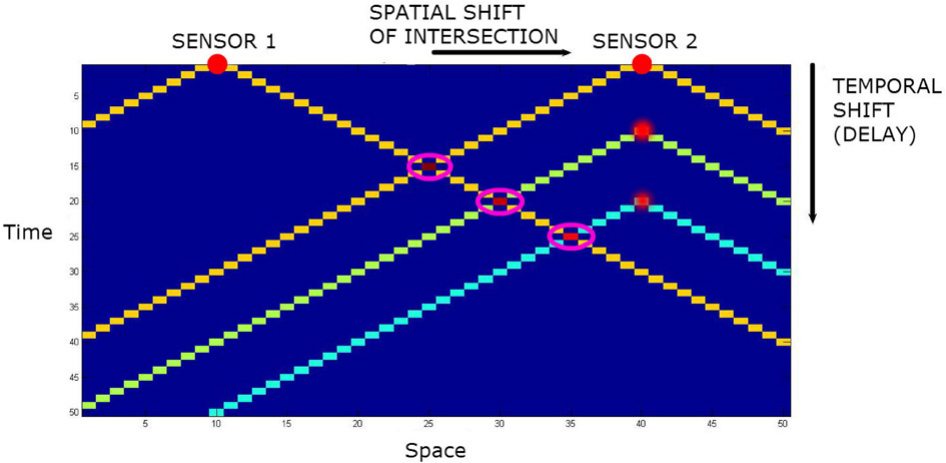
**The spatiotemporal line of sight of two audio sensors in one spatial dimension plus time**. The intersections are highlighted by magenta rings. With no delay, the intersection of the lines of sight is the point equidistant from the two sensors. By adding temporal delays to one sensor, the intersection can be translated in space.

Let us add audio capacity to the agents. The “mouth” is located midway between the “eyes” of the agent, and generates an audio event at each time step. The omnidirectional “ears” are co-located with the “eyes.” However, they have a different LoS. The momentary LoS *L*_Ear_ of an ear may be defined as a subset of *W* wherein the spatial distance from source to sensor equals the temporal distance from source to sensor. This takes the form of a cone in spacetime. See Figure 4. Assuming the sensor is at the origin and sound travels at one spatial quanta per temporal quanta, this is just;

(9) $$\begin{array}{l} \;L_{\text{Ear}}\subset W\\ \forall w\{w\in L_{\text{Ear}}:\tau_{w}=-\rho_{w}\}. \end{array}$$

The aural LoS are integrated in just the same way as the visual LoS;

(10) $${\dot{S}}_{\text{Ears}}=\&(\vee L_{\text{Ear1}},\vee L_{\text{Ear2}}).$$

Ignoring time, the intersection *L*_Ear1_ ∩ *L*_Eye2_ forms a straight line in space defined by equidistance from both sensors. See Figure 3. This is equivalent to the “beam” of directional tuning projected in beamforming (Figure 1). To avoid the inefficiency of simulating sound propagation, the code used for the current model implements audio beamforming by hand. For each sound source, the code directly measures its offset from the auditory midline *L*_Ear1_ ∩ *L*_Eye2_. This value can then be used to generate a motor command for auditory orienting as in Equation (12). Naturally, the real world case with reverberations etc. becomes much more complex, but the simplified implementation is sufficient for demonstrative purposes.

#### 3.2.7. Intermodal integration

We now introduce an intermodal meta-sensor $\ddot{S}$, which combines the signals from the two unimodal meta-sensors *Ṡ*_Ears_ and *Ṡ*_Eyes,Center_, again with an AND gate. This operation may be described in full as;

(11) $$\ddot{S}=\&(\text{\&}(\vee L_{\text{Ear1}},\vee L_{\text{Ear2}}),\text{\&}(\vee L_{\text{Eye1},\text{Center}},\vee L_{\text{Eye2},\text{Center}})).$$

$\ddot{S}$ = 1 if and only if all the lines of sight of the agent's individual sensors intersect *W*_*C*_ (or equivalent in the appropriate modality e.g., *W*_*A*_ for audio events). Whether they intersect at the *same point* in *W*_*C*_/*W*_*A*_ or different points corresponds to the difference between location tuning and configuration tuning mentioned in “Visual beamforming” above. In terms of the momentary state of the agent's sensory apparatus, however, these different ecological situations are indistinguishable. The AND integration steps cannot decrease sparsity. Sparsity here corresponds directly to “selectiveness” in selective attention; with respect to sparsity/selectivity, $\ddot{S}$ ≥ *Ṡ*_SensorPair_ ≥ *S*_Sensor_.

Thus we arrive at an increasingly exclusive, hierarchical sensory definition of “like-me-ness,” in a formal sense amenable to implementation on a robot. The higher a momentary sensory sample $\ddot{L}$ reaches in the bilateral, multimodal hierarchy, topped here by $\ddot{S}$, the more attention it receives.

#### 3.2.8. Movement

We place one agent, representing the “subject” at the origin. This agent can only rotate on the spot. The other agent, representing the “stimulus,” is positioned at a depth of 50 quanta and a randomized position in Θ. It does not move. The stimulus is always oriented toward the subject at the origin, except where “averted gaze” is the experimental condition. See Figure 3. The default “searching” behavior for the subject is to rotate in a circle. If in the current timestep, *Ṡ*_Eyes,*i*_ = 1, anywhere in the visual field, the agent changes to an “align” behavior. When the subject is in align mode, it orients so as to center this stimulation by producing a motor command proportional to the offset of the correlated sensor pair from the center of the visual field. In the simulations where the ears and mouth are used, the subject also rotates to minimize the delay δ_Audio_ between the audio streams.

(12) $$\Delta \Theta =\eta (i_{\text{center}}-i_{\text{correlated}})+\eta \delta_{\text{Audio}}$$

where η is a motor gain constant for which we used η = −0.15σ (σ denotes sampling resolution). Together with the sensory bias for the center mentioned in the previous subsection, this “orienting-to-center” effectively implements an *a priori* motor assumption of special status for the midline. This “center is special” assumption probably deserves closer scrutiny in terms of its biological basis and potential justification in terms of optimal sampling strategies, but this topic is beyond the scope of the current paper.

## 4. Agent-based simulations

In this section, we describe a series of simulations based in the simple world just described. The simulations address minimal analogs of three newborn abilities; perception of size constancy (Granrud, 1987; Slater et al., 1990), visual face perception (Goren et al., 1975; Johnson et al., 1991) and audio–visual alignment (Guellai and Streri, 2011). In each of the simulations here, the “task” for the subject is to find and align with the stimulus, despite increasing levels of distractors which we place into the world. Distractors are randomly dispersed visual events which are permanent and immobile throughout the course of a trial. Where audio distractors are also used, each visual distractor may, with a 25% chance at each timestep, also generate an audio event. As a quantitative measure of performance, we recorded the angular distance in Θ of the subject from alignment with the stimulus at the end of the trial, and term this value the “error.” Each simulation was run 100 times and results averaged over all runs.

### 4.1. Simulation 1.0—size constancy

Granrud (1987) and Slater et al. (1990) found that newborns could perceive “size constancy” across changes in depth. Familiarization studies showed that the subjects could perceive a distinction between a patterned object and a double-sized (otherwise identical) object at double the distance, such that the size and form of the retinal projection were identical. The size constancy effect has been cited as some of the most convincing evidence for innate perceptual predispositions (Spelke, 1998; Streri et al., 2013b).

#### 4.1.1. Results

**Figure 5:**
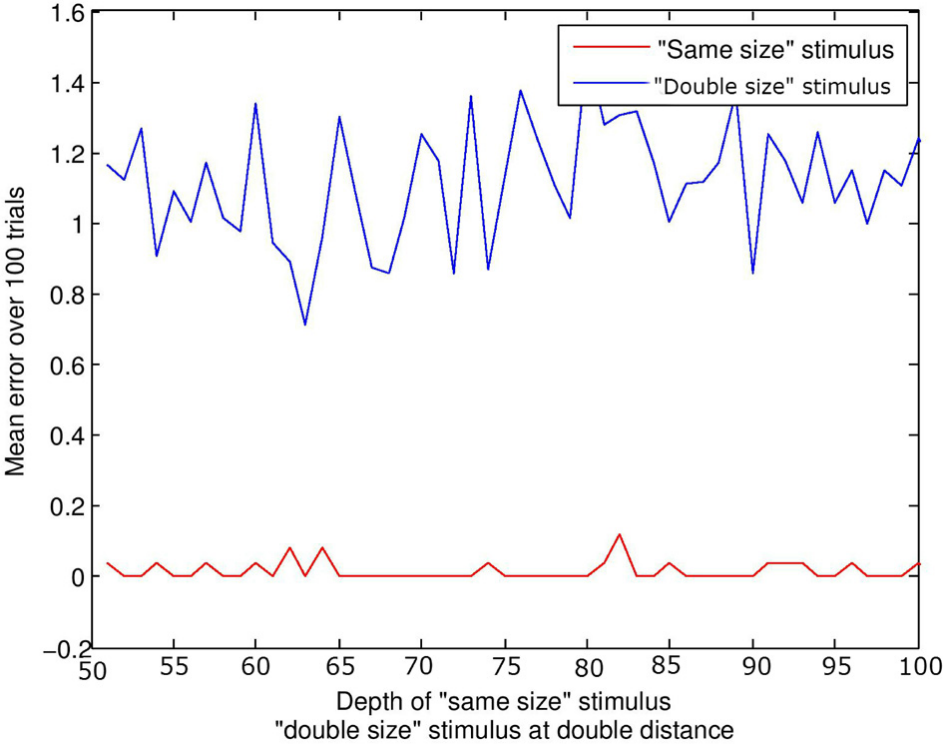
**Size constancy over depth**. The lines represents the error at the end of the trial. The red line represents the “same size” condition, and the blue line the “double size” condition. For a given depth *d* of the “same size” stimulus, the “double size” stimulus was at twice the distance 2*d*. Regardless of depth, the subject always finds and aligns with the “same size” stimulus, and ignores the “double size” stimulus.

This simulation did not include audio capacity or events. A stimulus of equal size to the subject was compared to a stimulus of twice the size. For the “same size” condition, the depth ρ of the stimulus was increased in increments of 1 quanta from 50 to 100 quanta. In the “double size” condition, depth increased in increments of 2 from 100 to 200 quanta; thus double the associated distance for the “same size” stimulus. Figure 5 displays mean error against distance for the two stimulus conditions. The distance of the stimulus from the subject makes little difference to the result; it is physical size of the configuration which is targeted by visual beamforming, and translation in depth makes no difference with parallel visual axes (until the limits of resolution are reached). See Figure 3. The subject finds the equal-size stimulus effectively, and ignores the double-sized stimulus equally effectively.

This is of course a special case; the current model would not discriminate a double-sized from a triple-sized stimulus, as it would ignore both. More generally, otherwise identical stimuli of different physical size may project in an identical manner to one retina if depth compensates, but may project differently in terms of the spatial relations between the images on *both* retinae. Note that disparity sensitivity is not a prerequisite; interactions between corresponding points may in many cases be sufficient.

#### 4.1.2. Discussion

Streri et al. (2013b), after Holway and Boring (1941), state that in order to recognize size constancy, infants need to combine projective size with information about viewing distance. Neither of these values are used explicitly in the current model, which nonetheless exhibits a size constancy effect. Perception of size constancy implies that the stimuli must be characterized by some difference in the way they impact the senses. If there were no detectable difference between stimuli at all, the behavioral distinction would be not just innate, but supernatural. If there is a sensory difference, then a familiarization effect does not imply any specific functional adaptation. Binocular correlation patterns could provide an informational basis for the discrimation between “small” and “far away” in the newborn. Monocular motion parallax might provide another potential source of the distinction.

#### 4.1.3. Prediction

Size constancy in newborns will be lost under monocular viewing conditions. This would distinguish the potential contributions of binocular and monocular mechanisms.

### 4.2. Simulation 2.0—face detection

Goren et al. (1975) and Johnson et al. (1991) found evidence for an innate preference for face-like stimuli. We recently showed that these results might result from a newborn “preference” for binocular correlations (Wilkinson et al., in press). Existing theories of newborn face preference posit some kind of monocular neural filter applied to the retinal image (Morton and Johnson, 1991; Turati, 2004; Pitti et al., 2013).

#### 4.2.1. Results—binocular correlations versus monocular template matching

**Figure 6:**
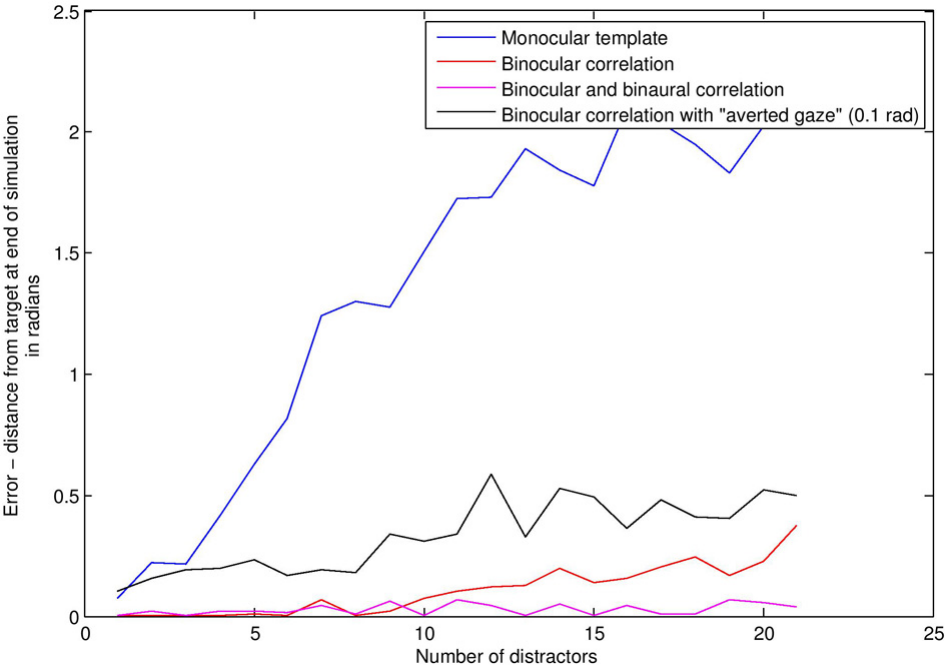
**Face detection error rates (vertical axis) under increasing levels of distractors (horizontal axis)**.

**Figure 7:**
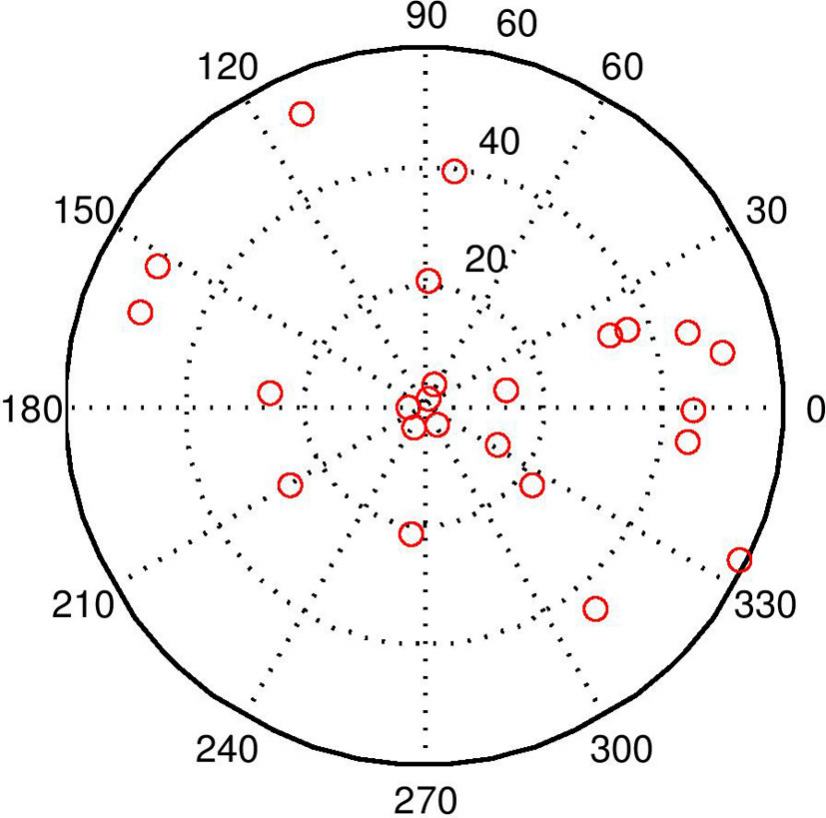
**This image shows the world with 20 distractors**. The task is quite difficult under these conditions, but the BCM does a reasonable job even with visual beamforming only. With audio–visual beamforming, performance is excellent even with high levels of distractors.

We compared a subject using bilateral gain control with a subject using a “face template” monocularly in each eye. This template consists simply in seeing two or more visual signals at the same time, analogous to applying a “two blob” template across spatial scales. If such a situation occurs, the template subject rotates to center the position of the match, averaged between the two eyes. There is no audio in this first simulation. Figure 6 depicts average angular distance of the subject from the stimulus on the vertical axis, against increasing levels of distractors on the horizontal axis. Without distractors, finding the stimulus is a trivial matter of finding the only stimulus around, and so both BCM (red line) and template (blue line) methods perform very well. With high levels of distractors (Figure 7), the task is really quite difficult. As distractors are added and *selective attention* is required, the BCM subject substantially outperforms the template subject. This is because the BCM subject is tuned to a particular physical size of stimulus regardless of depth, whereas the monocular template subject confounds size with depth, and is therefore more prone to distractors. Confounding depth with size is a general feature of templates which match the monocular visual array.

#### 4.2.2. Results—averted gaze

Studies have shown that good visual alignment of the stimulus with the subject (“direct gaze”) plays an important role in newborn face perception (Farroni et al., 2002; Guellai and Streri, 2011). To model these findings, we alter the alignment of the stimulus, such that it no longer “looks” directly at the subject, to provide a minimal analog of what is termed in the literature “averted gaze.” We examined the effect of averted gaze on face detection, using the binocular correlation based detector (black line in Figure 6). The result is a roughly constant increase in the error rate over all distractor levels. This arises because although the subject is often able to find the averted gaze stimulus, it is unable to align precisely with it, and so oscillates around the stimulus instead of coming to rest at perfect alignment.

#### 4.2.3. Results—audio-visual face detection

Adding the audio capabilities described in Audio Sensing and Signalling enables improvements in face detection, because $\ddot{S}$ is more selective than *Ṡ*_Eyes_ (see “Intermodal integration” above). Audio distractors were used in this simulation. The stimulus was always in the “direct gaze” condition. The magenta line in Figure 6 shows the error with audio-visual beamforming. Even with high levels of distractors (Figure 7), the stimulus is usually located effectively; a configuration of audio and visual distractors matching the form of the stimulus is quite rare, even with up to 20 distractors.

#### 4.2.4. Discussion

This minimal model demonstrates “like me” detection through audio-visual beamforming, in a manner which is highly dependent on good mutual alignment. The astute reader may note that in the real world, babies have different inter-pupillary distance (“IPD”) to their adult conspecifics. This is indeed an important factor. We have previously shown that this need not be a major problem in the real world case, as the spatial extension of the signal from the eyes can compensate for some difference in IPD (Wilkinson et al., in press). In general, allometry and alignment are relevant to the behavioral outcomes of bilateral gain control, and will generate individual and inter-trial differences. To keep things simple, we do not expand on this topic in the current article.

#### 4.2.5. Prediction

Newborns will prefer stimuli which induce strong binocular correlations over monocular contrast matched controls which do not. A certain level of “face preference” will drop out of this more general effect.

### 4.3. Simulation 3.0 face-voice integration: the role of direct gaze

Sai (2005) elegantly demonstrated that newborns identify their mothers face via the association of her voice, which they already recognize from the prenatal period. In recent extensions of this work, subjects showed long term recognition effects for dynamic faces only when accompanied by speech, supporting an important role for multimodal stimulation in triggering/gating learning (Guellai et al., 2011). Beyond this, the faces had to fixate the infant; averted gaze abolished recognition effects (Guellai and Streri, 2011). Here we asked whether audio-visual beamforming would produce analogous dependencies on good alignment between subject and stimulus.

In this simulation, the stimulus either aligned properly with the subject—the “direct gaze” condition—or is rotated out of alignment—the “averted gaze” condition. The amount of rotation was incrementally increased between 0.0 and 0.2 radians. We do not model learning *per se* in the current contribution, as would be required to model recognition effects completely. Instead, we constrain the discussion to selective attention, on the basis that attention defines what is learned. Learning is assumed to occur if and only if $\ddot{S}$ = 1. We then measured the average (over 100 simulations) amount of time the subject spends in “learning mode” (i.e., $\ddot{S}$ = 1) under each increasing misalignment of the stimulus. There were no distractors in this simulation.

#### 4.3.1. Results

**Figure 8:**
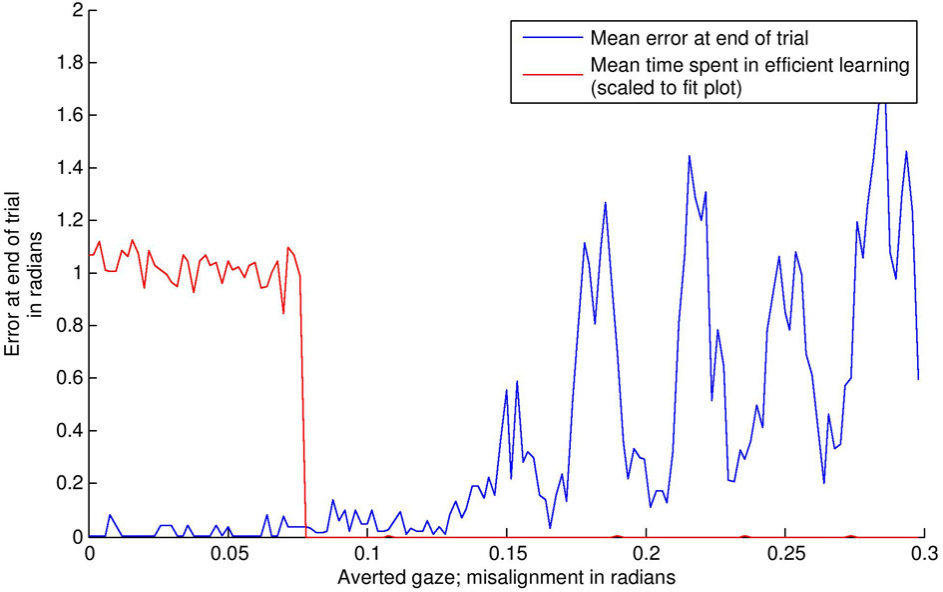
**The effects of averted gaze on attention and learning**. The blue line shows mean error (over 100 trials) versus level of misalignment. The red line shows the mean amount of time spent usefully in “learning mode” (i.e., $\ddot{S}$ = 1, and the subject has the stimulus in view). These latter data have been scaled to fit on the graph. Misalignments up to about 0.07 radians are mostly undetectable, and the subjects spend about half the trial on average (the expected mean amount if the subject usually finds the stimulus) in learning mode, with the stimulus in their field of vision. Over this level, there is a steep drop off in time spent learning. Over about 0.11 radians, the subject's ability to locate the stimulus at all becomes increasingly poor.

The results of this simulation are displayed in Figure 8. In the “direct gaze” condition (0.0 radians), the subject finds the stimulus and aligns with it effectively. As a result, it goes into “learning mode” (i.e., $\ddot{S}$ = 1) for the remainder of the simulation. Small misalignments up to about 0.07 radians are pretty much undetectable at the resolution used here. Over this level, the amount of time spent learning tends quickly to zero. This is because the subject finds the stimulus, but then oscillates rapidly around it, trying but failing to align with both the audio and visual signals. As misalignment increases, the subject ceases to locate the stimulus effectively.

**Figure 9:**
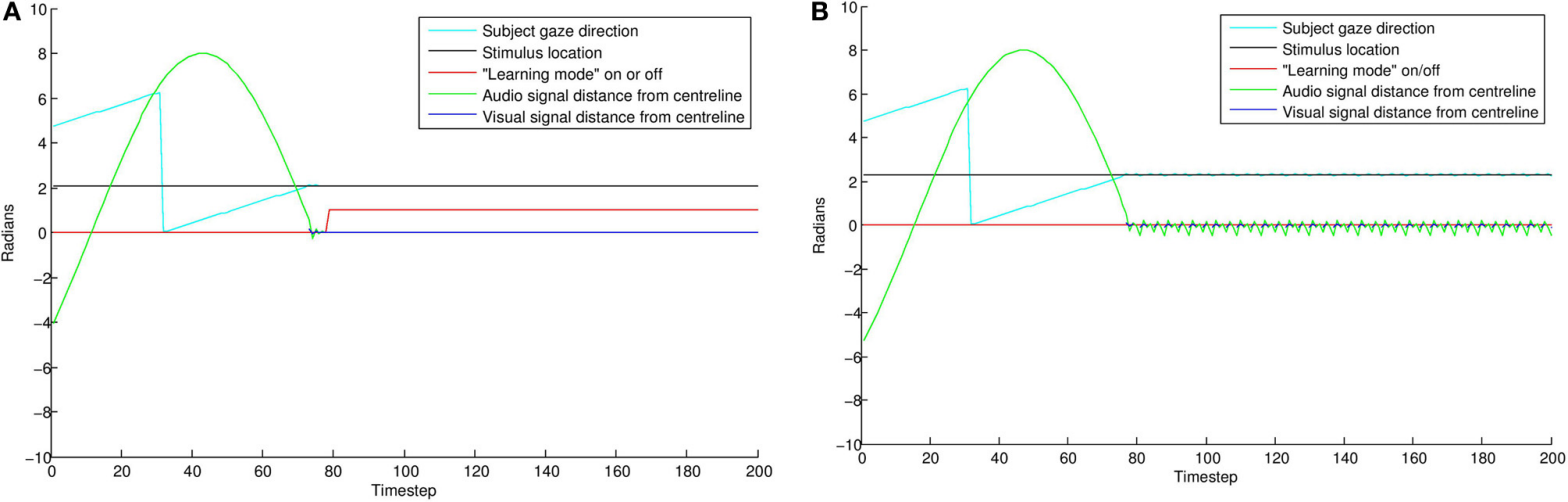
**Comparing individual simulations with “direct gaze” (A) and “averted gaze” (B) by 0.08 radians**. With a direct gaze stimulus, the subject (cyan line) finds the stimulus (black line) and quickly aligns until error is zero. It then goes into learning mode (red line) for the rest of the trial. With the averted gaze stimulus, the subject finds the stimulus, but is unable to align precisely with it. This results in the oscillatory aligning movements visible in the gaze direction and auditory signal (green line). As a result of the failure to align precisely, the subject does not go into learning mode. See Figure 10 for a zoomed in view of these oscillations.

**Figure 10:**
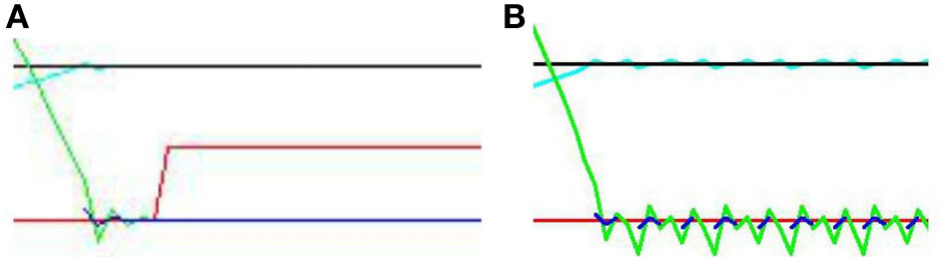
**Close up of Figure 9 (A) “direct gaze” and (B) “averted gaze” by 0.08 radians**. In the direct gaze condition, the audio signal (green line) aligns perfectly with the midline. The visual signal (blue line) does too. Hence “learning mode” (red line) goes on. In the averted gaze condition, this alignment fails to converge. The subject continues to oscillate, attempting to align with both the visual and auditory signals. The audio (green line) and visual (broken blue lines) signals oscillate around good alignment without ever both achieving it. Thus the subject never enters learning mode.

Figure 9 displays results from two individual trials. In the direct gaze condition Figure 9A, both the audio and visual transceivers of the subject quickly align perfectly with those of the stimulus. With gaze averted by 0.1 radians Figure 9B, the subject tries unsuccessfully to align with the stimulus, resulting in a fast oscillation around the stimulus. The green line represents the distance from synchrony of the binaural time signals. Note the fast oscillation, generated by the oscillation of the subject around the stimulus (Figure 10). Averted gaze means the subject cannot align properly with both the visual signal (broken blue line) and the audio signal at the same time, and so $\ddot{S}$ = 0 at all times. “Learning mode” is obviously an oversimplification, but infants may be suffering from similar difficulty in aligning their sensor arrays with misaligned stimuli.

#### 4.3.2. Discussion

These simulations show that bilateral gain control can in principle generate a strong dependence on alignment of multimodal signal sources. The beamforming technique projects the tuning of the sensor array to a particular subvolume of physical and configural space. A set of unimodal specifications combine to build up a multimodal attentional “template,” which is projected onto the world as the combined multimodal “beams” of the array. This projected template describes an “ideal stimulus” in terms of what modalities of signal should be where and in what relations *in physical space*. The establishment and success of an interaction depends upon the extent and accuracy to which this set of selective conditions are met.

We are implementing a slightly more sophisticated model of the BCM on the humanoid robot iCub. The robot's visual system consists of monocular spatiotemporal filters in a simple *predictive coding* architecture (Rao and Ballard, 1999), based on *adaptive pattern generators* (Righetti and Ijspeert, 2006). Sensory input is subjected to an *active cancelation* through negative feedback of the learned signal. Essentially this is a minimal implementation of sensory adaptation; see Lieder et al. (2013) for a more realistic example of predictive coding applied to adaptation. The remaining sensory input is then subjected to bilateral mutual gain control. Audition uses purely temporal filters, but is otherwise based on the same mechanisms. It is worth noting that the existing literature on *active cancelation* (Kuo and Morgan, 1999) might provide a useful bridge between predictive coding [in particular the influential *active inference* formulation (Friston, 2010)], and the radically embodied implications of array sensing.

Attention is expressed through saccadic eye movements and neck movements, so as to centralize the source of the strongest region of post filter energy in the visual field, using the existing iCub oculomotor system. Vision is blocked during these movements. If at any given time, a “strong” (defined by some threshold) post-filter visual signal exists in the central region of vision, *and* is accompanied by strong binaural correlation (analogous to $\ddot{S}$ = 1), then the robot enters an “attentive” state in which it records the ensuing audio-visual stream to disk. A value representing this state is incremented whenever the above conditions are met, and otherwise decays slowly. We will then analyze the recorded periods, and assess their selectivity and reliability with respect to identifying the “interaction events” we instigate during the trial. This is obviously a much simplified model of the newborn case, but may still be instructive. The “social” functionality is anticipated partly because humans form the bulk of unpredictably moving objects in the lab (and a newborn babies world), and partly because their transceiver array is the right size and shape to cause bilateral correlations in multiple modalities (in a humanoid robot). We believe that identifying and carefully characterizing such ecological factors, together with defining minimal, biologically plausible mechanisms to exploit them, is crucial both to understanding how basic, naïve social attention is apparently so *easy* for babies, and to giving robots a similar “social instinct.”

#### 4.3.3. Predictions

1. In the psychophysical experiments detailed in Guellai and Streri (2011), “averted gaze” interfered with recognition effects. The BCM predicts that in a reproduction of this experiment, “averted voice” (or binaural misalignment of the sound source for the voice) will also impair recognition effects. Stereo speakers with delays could be used to manipulate the apparent source location of the voice in a subtle, non-intrusive way.
2. We predict that, in the cluttered, busy laboratory environment, the robot described above will preferentially record to disk during “social interaction” events, in particular the case where an interaction partner visually aligns with, and talks to, the robot (which people in the lab will be asked to do). It will occasionally record other events which just happen to impact the senses in the right way to pass the filter. We will also compare audio-visual beamforming against visual beamforming alone, and against mono mechanisms alone, in order to isolate the contributions of mono spatiotemporal filters and predictive coding, multi-modal coincidence, and bilateral mutual gain control.

## 5. General discussion

Bilaterality provides a spatially selective supramodal dimension along which to collapse the spatial and modal extent of the sensor array. Bilateral mutual gain control can enable spatial-configural tuning through beamforming. The existing evidence that bilateral gain control gates sensory flow in adults of various species practically implies a downstream effect on the assortment of observable outcomes collectively termed “attention” (Li and Ebner, 2006; Ding et al., 2013; Wunderle et al., 2013; Xiong et al., 2013). The major open questions with respect to “innate” perception, attention, and sociality are the nature of bilateral interactions in the human neonate and the implications of this physiological organization on behavioral and ontogenic timescales. The former can be addressed experimentally in neonates, while the latter can be explored via longitudinal empirical designs, mathematical modeling and developmental robotics (Morse et al., 2011).

The formal model and agent based simulations presented here are intended in a purely pedagogical sense, to specify and clarify our argument and demonstrate its consequences, and provide no evidence that newborn perception employs similar mechanisms. However, as for any useful theory, the current proposal *does* link back to the biological case via the predictions it makes regarding newborn behavior. Given knowledge of a newborn subject's bilateral sensory perspectives, a BCM based model can make real time, individual level salience predictions, with a precision that depends on the quality of perspective reproduction and the physiological detail of the model. If testing confirms these predictions, then the BCM will provide a powerful tool to understand early perceptual and social development.

The fundamental generative prediction of the bilateral correlation model of newborn attention is that the spatial-configural attentional biases of newborn babies will be a function of the extent to which multimodal contrast energy in the scene projects synchronously to bilaterally corresponding points on the subjects sensor array, given whatever mono filters are in use. Many experimental paradigms are possible, but the classical preference-on-average over a population of subjects must be replaced by a paradigm focussed at the individual level, as the BCM makes predictions which are dependent on the real time allometry and alignment of the subject's sensor array. Stereo source manipulation of the auditory “sweet spot” offers an unintrusive stimulus paradigm for assessing the role of binaural correlation in newborn attention and learning. Screen based eye-tracking could enable the use of dynamic, responsive stimuli which can manipulate binocular correlation in real time for individual subjects.

Our perspective is substantially in agreement with theories that innate sociality is based on perceptuomotor resonance and motor contagion (Meltzoff and Decety, 2003; Blakemore and Frith, 2005; Lepage and Théoret, 2007; Sugita, 2009; Pitti et al., 2013). However, the BCM differs in taking more seriously the role of the body, and resulting sensory and ecological geometry, in which the brain and its activity are situated. Innate knowledge of the “like me” is embedded in the sampling biases implied by sensory morphology and behavioral repertoire, inviting a broader conception of the “mirror organism.” The shared anatomical structure of interacting brains facilitates the interpersonal synchronization of brain activity (Dumas et al., 2012). Beamforming provides a mechanism by which shared corporal embodiment can play a similar role in mediating spatiotemporal alignment between interactors. It has been suggested that the “social brain network” and the “resting state network” may substantially overlap (Schilbach et al., 2008). The current model carries a similar message at the corporal level; bilateral sensor distribution and integration can create a sampling bias for the “like me” which frames neural and bodily development in a deeply and intrinsically social context from the earliest stages of sensory sampling and the beginnings of experience in the world. We suggest that the current proposal is best interpreted as a contribution to the recent literature developing an *enactive* theory of early social development (Varela et al., 1991; O'Regan and Noë, 2001; De Jaegher and Di Paolo, 2007; Auvray et al., 2009; De Jaegher et al., 2010; Di Paolo et al., 2010; Lenay et al., 2011; Di Paolo and De Jaegher, 2012; Froese et al., 2012; Lenay and Stewart, 2012).

The BCM is relatively easy to implement on a robot, at least in the simple “newborn” form presented here. The basic requirements are a bilaterally organized sensor array geometry, and an integration step based on mutual gain control. The nature of an audio or visual “event” in the current model is deliberately abstract. In the real world case, the choice of sensors and the mono filters applied will define what qualifies as an event. In the case of continuous valued sensor data, the binary AND used for intersensory integration may be replaced by multiplication or a more complicated gain control function, perhaps including normalization. The behavioral outcomes which emerge will depend on the form of the bilateral sensor pair's LoS and the bilateral morphology of the sensor array, shifting much of the explanatory burden for observable functional specificity to the embodiment of the agent and the ecological context. Therefore the design of the robot's sensor array becomes crucial to behavioral outcomes. Equally, this implies the natural selection can mould behavioral outcomes by moulding the phenotypic instantiation of sensor array morphology. Note for example the relatively large head and inter-pupillary distance of the human newborn (Pryor, 1969), which brings the newborn sensor array closer to good spatial tuning with that of adult conspecifics. This may be a candidate for a specific adaptation for newborn social interaction, though may also reflect other necessities such as a large brain.

The BCM is in agreement with current gain control models of bilateral integration (e.g., Ding et al., 2013) and interfaces cleanly with existing models attention based on gain modulation (Hillyard et al., 1998; Salinas and Sejnowski, 2001; Aston-Jones and Cohen, 2005; Reynolds and Heeger, 2009; Feldman and Friston, 2010; Sara and Bouret, 2012), spatiotemporal energy models of early vision (Mante and Carandini, 2005), and computational methods based on salience mapping (Itti and Koch, 2001), wherein it can simply provide another contributor to overall salience. Predictive coding (Rao and Ballard, 1999; Bastos et al., 2012) and its generalization in the free energy minimization framework (Friston, 2010; Moran et al., 2013) are becoming increasingly influential as models of brain function. In this approach, a major factor influencing gain modulation is the *predictability* of the signal (Feldman and Friston, 2010). Models based on “artificial curiosity” or “intrinsic motivation” (Barto, 2013; Gottlieb et al., 2013) take a similar approach. Bilateral mutual gain control is more *a priori*, in that modulation is [ignoring potential interaction through bilateral normalization (Moradi and Heeger, 2009)], *independent* of the modulated signal. Bilateral mutual gain control is also (we found) awkward to formalize and justify in terms of predictive coding.

In terms of the information compression analogy, bilateral mutual gain control maps more comfortably to an embodied form of lossy *transform coding*, wherein the basis function in which a mono signal is recoded is simply (if unconventionally) the signal from its stereo twin, and vice versa. The application of lossy transform coding followed by lossless predictive coding is standard practice in data compression (Clarke, 1995). The role of transform coding is to order information in a format where threshold based mechanisms can most effectively eliminate information according to some *a priori* value function. For example, in MP3, the audio time signal is subjected to Fourier transform and components which are undetectable to a computational model of human hearing are discarded. The functional justification is reduced file size with little appreciable loss of sound quality for human listeners. In the BCM, signals which are not part of multimodal configurations “resonant” with the allometry and alignment of the array—quantified in terms of bilateral correlation—are discarded/damped. The transform consists in the large scale neural morpho-architecture collapsing the space between bilateral sensor pairs. The sharpness of the filter's cut-off may be manipulated by mono resolution and by allowing a certain extent of spatiotemporal cross-correlation as well as pure correspondence. The functional justification is selective spatial-configural tuning to the “like me,” in the broadest sense of the term.

The combination of local (mono) decorrelation, followed by global (bilateral) correlation, may turn out to be justified on sparsity principles (Vinje and Gallant, 2000), and possibly on free-energy minimization principles (Feldman and Friston, 2010) as priors expressing the expectation that spatially neighboring locations in the world are likely to be correlated, whereas spatially distributed locations in the world are not. The thermodynamic unlikeliness of corporal form ensures that it is rarely approximated by random happenings, providing selectivity, while heredity validates the assumption that resonant signals are likely to be interesting, providing relevance.

The principle of beamforming through mutual gain control is not limited to the example of bilateral symmetry expanded here. An interesting and testable, if speculative, prediction is that a mutual gain control relation will exist between “corresponding points” on the fingertips of a single hand, across all age ranges. Work on this topic could help us to understand naïve and skilled use of the hand as a sensor array to apprehend the ecological geometry and dynamics (Gibson, 1961, 1966, 1977), and resulting sensorimotor contingencies (O'Regan and Noë, 2001), of interacting with different shapes, densities, and textures.

### 5.1. Conclusion

We have sketched an uncontraversial argument for bilateral mutual gain control as a basic perceptual mechanism. Bilateral mutual gain control offers an effective and efficient multimodal “like me detector” available to both biological and robotic systems. Some form of bilateral interaction in newborns is much more likely than no bilateral interaction given current evidence, and (perhaps immature) mutual gain control is the strongest candidate for integration. Perhaps more contraversially, we argue that this may explain a number of “innate predispositions” observed in newborn infants. Bilateral interactions have been largely ignored as potential causes of innate predispositions in the developmental psychology literature. Whilst we agree to a large extent with the notion of the competent newborn, the current paper is aimed to target questionable adaptationist, nativist, and internalist assumptions regarding causal structure. Empirical work is required to establish the nature of bilateral interaction in the newborn human, and its potential relevance to particular abilities observed in newborns. Though the current model is a greatly simplified one, the mechanisms involved are very general. Ongoing work is extending the model to dynamic sensor distribution through behavior, simple perceptual learning/adaptation through predictive coding, interactive scenarios, and real world embodiment on the humanoid robot iCub. Whether bilateral mutual gain control can account, fully or partially, for particular existing empirical results is a case-by-case question, and we certainly do not want to assert that the BCM can fully explain newborn social skills. However, we do wish to push the point that *ignoring* bilateral integration has led to questionable interpretations of newborn abilities. In summary, the effects of bilateral integration should be considered and controlled for in the planning, execution, and interpretation of psychophysical experiments investigating perception, attention, and sociality in newborns.

### Conflict of interest statement

The authors declare that the research was conducted in the absence of any commercial or financial relationships that could be construed as a potential conflict of interest.

## Footnotes

^1^http://www.jach.hawaii.edu/JCMT/continuum/scuba2.html
